# Learning “graph-mer” Motifs that Predict Gene Expression Trajectories in Development

**DOI:** 10.1371/journal.pcbi.1000761

**Published:** 2010-04-29

**Authors:** Xuejing Li, Casandra Panea, Chris H. Wiggins, Valerie Reinke, Christina Leslie

**Affiliations:** 1 Department of Physics, Columbia University, New York, New York, United States of America; 2 Department of Genetics, Yale University, New Haven, Connecticut, United States of America; 3 Department of Applied Physics and Applied Mathematics, Columbia University, New York, New York, United States of America; 4 Computational Biology Program, Sloan-Kettering Institute, New York, New York, United States of America; Broad Institute of MIT and Harvard, United States of America

## Abstract

A key problem in understanding transcriptional regulatory networks is deciphering what *cis* regulatory logic is encoded in gene promoter sequences and how this sequence information maps to expression. A typical computational approach to this problem involves clustering genes by their expression profiles and then searching for overrepresented motifs in the promoter sequences of genes in a cluster. However, genes with similar expression profiles may be controlled by distinct regulatory programs. Moreover, if many gene expression profiles in a data set are highly correlated, as in the case of whole organism developmental time series, it may be difficult to resolve fine-grained clusters in the first place. We present a predictive framework for modeling the natural flow of information, from promoter sequence to expression, to learn *cis* regulatory motifs and characterize gene expression patterns in developmental time courses. We introduce a cluster-free algorithm based on a graph-regularized version of partial least squares (PLS) regression to learn sequence patterns—represented by graphs of *k*-mers, or “graph-mers”—that predict gene expression trajectories. Applying the approach to wildtype germline development in *Caenorhabditis elegans*, we found that the first and second latent PLS factors mapped to expression profiles for oocyte and sperm genes, respectively. We extracted both known and novel motifs from the graph-mers associated to these germline-specific patterns, including novel CG-rich motifs specific to oocyte genes. We found evidence supporting the functional relevance of these putative regulatory elements through analysis of positional bias, motif conservation and *in situ* gene expression. This study demonstrates that our regression model can learn biologically meaningful latent structure and identify potentially functional motifs from subtle developmental time course expression data.

## Author Summary

A major challenge in functional genomics is to decipher the gene regulatory networks operating in multi-cellular organisms, such as the nematode *C. elegans*. The expression level of a gene is controlled, to a great extent, by regulatory proteins called transcription factors that bind short motifs in the gene's promoter (regulatory region in the non-coding DNA). In a temporal regulatory process, for example in development, the “regulatory logic” of DNA motifs in the promoter largely determines the gene's expression trajectory, as the gene responds over time to changing concentrations of the transcription factors that control it. This study addresses the problem of learning DNA motifs that predict temporal expression profiles, using genomewide expression data from developmental time series in *C. elegans*. We developed a novel algorithm based on techniques from multivariate regression that sets up a correspondence between sequence patterns and expression trajectories. Sequence motifs are represented as graphs of sequence-similar *k*-length subsequences called “graph-mers”. By applying the method to germline development in *C. elegans*, we found both known and novel DNA motifs associated with oocyte and sperm genes.

## Introduction

The mRNA expression level of a gene is regulated by multiple input signals that are integrated by the *cis* regulatory logic encoded in the gene's promoter. Genes whose regulatory sequences contain similar DNA motifs are likely to have correlated expression profiles across a given set of experimental conditions. The converse, however, is not necessarily true. That is, genes can have correlated expression profiles without being coregulated, since multiple regulatory programs may lead to similar patterns of differential expression. This is particularly evident in developmental time series data, in which the genes exhibit only a few distinct expression patterns. Nevertheless, computational approaches for deciphering gene regulatory networks from gene expression and promoter sequence data often do assume that correlation implies coregulation. For example, a typical computational strategy is to cluster genes by their expression profiles and then apply motif discovery algorithms to the promoter sequences for each cluster. The cluster-first motif discovery approach is indeed so prevalent that the best-known benchmarking study of motif discovery algorithms [1] defines the problem in precisely this way – namely, given a cluster of genes, find the overrepresented motif(s) in the promoter sequences – and compares numerous such algorithms. It is clear, however, that assigning genes to static clusters that are assumed to be coregulated oversimplifies the biology of transcriptional regulation. Moreover, in a setting where there are few experiments probing the conditions of interest or where many genes have synchronized expression profiles, such as in a time course, clustering may fail to resolve meaningful gene sets for subsequent motif analysis.

In the current work, we present an algorithm that models the natural flow of information, from sequence to expression, to learn cis regulatory motifs and to characterize gene expression patterns. Our algorithm learns motifs that help to predict the full expression profiles of genes over a set of experiments, with no clustering. More precisely, we use a novel algorithm based on partial least squares (PLS) regression to learn a mapping from the set of 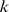-mers in a promoter to the expression profile of the gene across experiments; in time series, we learn 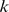-mers that help to predict the full expression time course for genes. PLS combines dimensionality reduction and regression; it iteratively finds latent factors in the input space with maximal covariance with projections in the output space. We introduce a graph-regularized version of the PLS algorithm to enable motif discovery by imposing two constraints: a lasso [2] constraint for sparsity and a graph Laplacian constraint for smoothness over sequence-similar motifs. Our novel graph-regularized PLS algorithm can be used in any situation where the input features are related by a graph structure. Here, the graph structure is defined on the feature space of 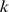-mers, with edges connecting pairs of similar 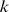-mers. Our approach is motivated by recent machine learning work that uses the graph Laplacian to exploit graph structure in various ways, for example, by defining a graph over training examples in semi-supervised classification (Laplacian SVM [3]) and clustering (spectral clustering [4]) as well as imposing graph smoothness on features of an SVM classifier [5].

Our focus in this study is discovering regulatory elements and deciphering transcriptional regulation in the nematode *Caenorhabditis elegans*, a key model organism in developmental biology. In particular, we are interested in using mRNA profiling experiments from developmental time courses, where the high global level of correlation presents a challenge to clustering. Dissection of gene regulatory logic is not as advanced in *C. elegans* as it is in *D. melanogaster*, for example. There are few motif discovery programs designed specifically for worms, and while worm biologists do use generic programs such as MEME [6], traditionally they have relied on experimental strategies to define binding motifs and then performed genome-wide motif searches and validation with transgene reporters. One goal of our work is to advance this area of inquiry by defining novel elements and providing new opportunities for directed experimental validation.

As a demonstration of our method, we applied our graph-regularized PLS algorithm to an expression time course for wildtype germline development in *C. elegans* [7]. We found that the first and second PLS latent factors mapped to expression profiles for oocyte and sperm genes, respectively. In each iteration of our approach, we learn sequence information in the form of a “graph-mer”, i.e. a graph where vertices are 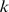-mers, weighted by their contribution to the latent factor, and edges join 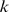-mers that are close in Hamming distance. To parse the motif graphs into component motifs, we applied a graph module discovery algorithm followed by hierarchical agglomeration to produce position specific scoring matrices (PSSMs) from the weighted 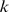-mers. Applying this procedure to the significant latent factors generated a collection of known and novel oocyte- and sperm-specific motifs, including novel CG-rich motifs associated with oocyte expression trajectories. One graph-mer derived sperm motif was a bHLH binding site motif and exhibited spatial bias in the promoters of sperm genes but not non-sperm genes. The functional relevance of the CG-rich motifs was supported by strong conservation between *C. elegans* and *C. briggsae* and was associated with germline-specific *in situ* expression patterns. This study gives an interesting proof of principle for using PLS regression models for transcriptional regulation in developmental time series.

## Results

### Learning graph-mer motifs and corresponding expression trajectories

In order to learn the correspondence between (sets of) regulatory motifs in the promoter sequences of genes and gene expression trajectories over a time course, we posed a regression problem: using a training set of 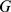 genes, learn a linear mapping from the vector of counts of 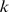-mer occurrences in a gene's promoter to the gene's time course expression profile. This model can then be used to predict expression from sequence on held-out genes, and 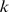-mer features that are highly weighted in the model should represent important regulatory motifs. Here we have a very high-dimensional input space of motifs (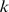-mers) as well as a multivariate output space, both of which rule out use of ordinary least squares regression. Instead, our algorithm makes use of a partial least squares (PLS) regression strategy. PLS is a well-known statistical technique for fitting linear models when the input space is high dimensional [8] and has both univariate and multivariate formulations.

Standard PLS represents the input data as a motif matrix **X** (dimension 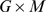, where 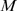 is the number of 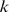-mers), representing 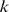-mer counts for each gene's promoter, and the gene expression matrix by **Y** (dimension 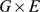, where 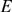 is the number of experiments), and then it performs two basic steps (see Methods for more details):

1. Construct 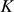 *weight* vectors 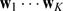 in 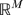 and corresponding *latent* factors 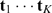 in 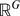, where the weight vectors are chosen so that the latent factors have maximal covariance with directions in **Y**. The latent factors define a reduced dimensional representation of the promoter sequence data.
2. Regress **Y** against the latent factors using ordinary least squares (or ridge) regression. The latent factor dimensionality reduction followed by linear mapping to **Y** yields the final mapping from sequence to expression.

PLS algorithms typically work iteratively, so that each round 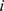 generates a new latent factor, and the number of rounds 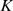 is chosen by cross-validation to minimize the square loss function in the regression problem.

**Figure 1:**
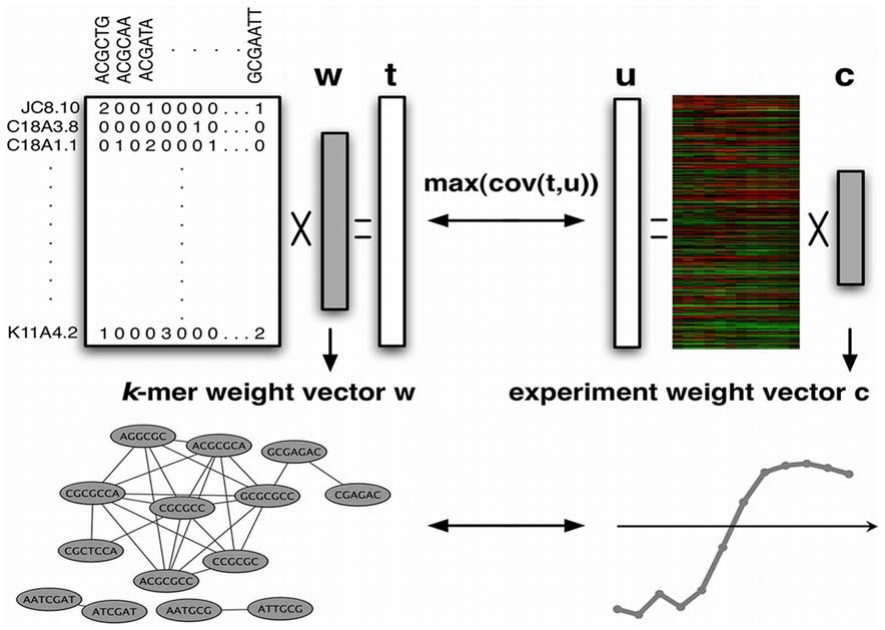
Mapping between motif weight vectors and experiment weight vectors. At each iteration 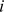 of the modified PLS algorithm, 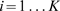, weight vectors 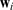 and 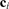 are derived by finding latent factors 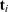 and 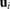 with maximal covariance. For clarity, subscripts 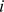 are omitted in the diagram and in the rest of the description. Each weight vector **w** is a vector in 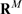, where 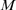 is the number of 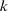-mers used as input to the algorithm. Due to graph-regularization, each weight vector is sparse, i.e. most 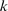-mers have weight 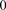, and smooth over a graph connecting sequence-similar 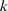-mers, i.e. similar 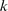-mers get assigned similar weights. Therefore, we can visualize the weight vector as a “graph-mer”, a graph where nodes correspond to 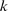-mers with high positive weights and edges connect sequence-similar 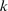-mers (bottom left). At each iteration, the PLS procedure sets up a correspondence between the motif weight vector **w** and a weight vector over expression experiments represented by vector **c**. In our setting, the series of expression experiments is a time course, and the vector **c** can be viewed as an expression pattern or trajectory (bottom right). Intuitively, we can think of the set of 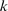-mers shown in the graph-mer as driving the expression pattern **c**. Roughly speaking, the model predicts that genes containing these 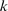-mers will have expression patterns that correlate with **c**; more precisely, the full regression model predicts gene expression patterns using all 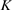 latent factors.

Here, we are most interested in what PLS tells us about the covariance structure between **X** and **Y** and how to interpret this information in terms of sequence motifs and expression patterns. In particular, along with 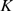 weight vectors 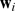 in the input motif space, PLS determines corresponding vectors 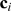 in the output expression space, defined so that 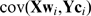 is maximal (Figure 1). Intuitively, each weight vector 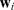 corresponds to a set of motifs (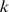-mers) that helps explain expression patterns in the direction 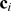. The components of the vector 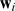 that have large positive weights are the 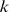-mers that most strongly predict the expression pattern 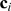.

To obtain a more interpretable model, we mathematically imposed two additional requirements on the PLS solution. First, we wanted the weight vectors 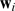 to be sparse, i.e. we wanted relatively few 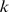-mers to have non-zero components, so that the algorithm produces a small number of hopefully functional motifs. Second, for each weight vector 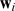, we wanted sequence-similar 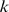-mers to have similar weights, since such 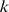-mers may represent variants of the same binding site and potentially should contribute in the same way to the linear model. We achieved the first goal by adding a lasso constraint to the PLS optimization problem (see Methods, equation (4)). For the second goal, we defined a graph on the set of 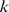-mers, joining two 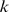-mers by an edge exactly when they are close in Hamming distance, and imposed a graph Laplacian constraint to obtain smoothness over the graph (see Methods, equation (7)). Incorporating these constraints into a multivariate PLS approach yields a new algorithm that we call graph-regularized PLS.

With these additional constraints, we can view the motif vectors 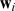 as “graph-mers” – weighted graphs over 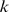-mers, where highly weighted dense clusters in the graphs correspond to important sequence-similar 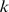-mer sets, or motifs. Figure 1 illustrates the mapping between motif weight vectors, interpreted as graph-mers, and corresponding expression patterns, arising from the latent factors found in graph-regularized PLS. Intuitively, we can think of each vector 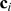 as the expression pattern driven by the positively weighted 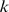-mers in 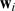, that is, the common expression trajectory displayed by genes containing these motifs. This correspondence will be important for interpreting regulatory motifs in worm germline development below.

### Graph-mer modeling for germline development in worm

We applied our graph-regularized PLS regression algorithm to time series gene expression data for wild-type germline development in worm *C. elegans* [7]. This data set consists of a time course beginning in the middle of the third larval stage (L3) and extending through adulthood. During this time, the major developmental changes occur in the germ line. Some germ cells undergo constant proliferation, while others initiate developmental events, including entry into meiosis followed by differentiation into sperm, which occurs in the fourth larval stage, or differentiation into oocytes, which occurs in young adults. By the end of the timecourse, animals have produced mature gametes and launched embryogenesis. Twelve samples were collected at 3-hour intervals with 3 replicates for each sample. Basic microarray data normalization was performed in the original study, and we used the normalized gene expression levels as reported (Gene Expression Omnibus, http://www.ncbi.nlm.nih.gov/geo/, accession numbers GSE726-GSE737). We averaged expression levels over replicates for 20,000 genes and calculated the 5% and 95% quantile of all expression values. We filtered out genes with baseline expression (defined here as having expression values between the 5% and 95% quantiles at all time points) and also ones that exhibit little variance in expression over time (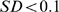). After further removing genes without upstream sequences from WormMart, we obtained the gene expression matrix for 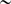
9,000 genes and 12 time points.

We downloaded promoter sequences spanning 500 bp upstream of transcription start sites from WormMart. For genes whose upstream intergenic sequence is shorter than 500 bps, we used the intergenic sequences instead of 500 bps upstream. We scanned the promoter sequences for candidate 6-mers and 7-mers, and filtered 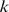-mers based on expected counts in background sequences (see Methods).

### Regularized PLS predicts held-out gene expression

**Figure 2:**
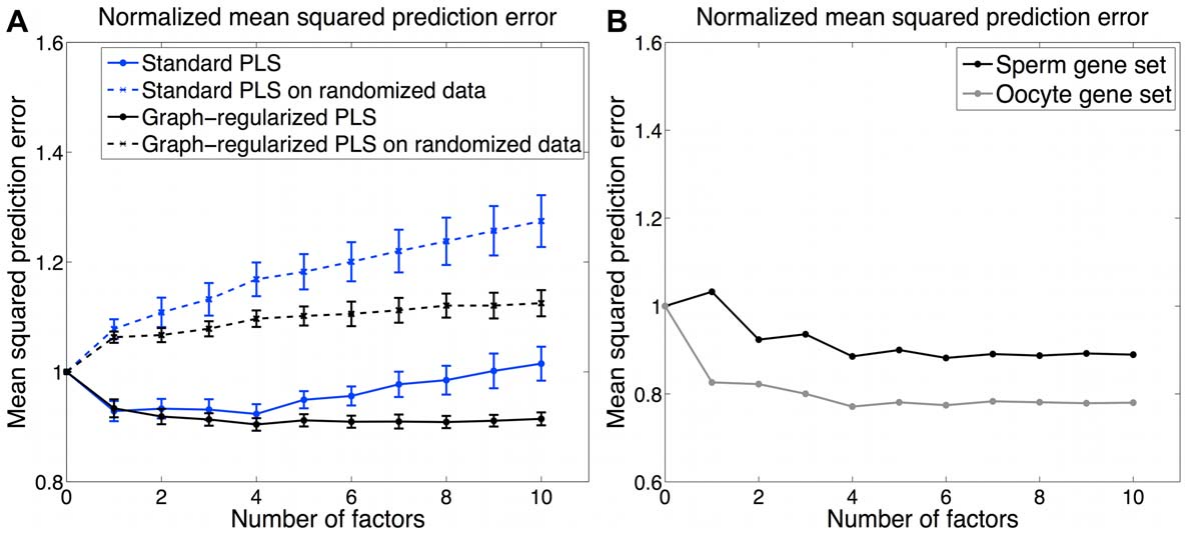
Normalized mean squared error on cross-validation test data. (A) Normalized mean squared error versus number of latent factors for standard PLS and graph-regularized PLS on real and randomized data. The mean squared error obtained with zero latent factor is normalized to 1. Computed standard deviations of squared error across cross-validation sets are plotted as error bars. For the real cross-validation data, standard PLS overfits after the 4th factor; graph-regularized PLS is more resistant to overfitting than standard PLS. As expected, when trained and tested on randomized data, both standard and graph-regularized PLS overfit with the very first factor. (B) Normalized mean squared error of sperm and oocyte gene sets for graph-regularized PLS. The first and second factors dominate oocyte and sperm genes respectively in terms of largest chi-square reduction.

We performed 10-fold cross-validation experiments, randomly splitting genes into test and training sets with 10% of the data assigned to test data. Figure 2A illustrates the normalized mean squared error (see Methods, equation (1)) on the cross-validation test sets versus number of latent factors for both standard and graph-regularized PLS. Here, the mean squared error obtained with zero latent factors (i.e. the variance of the test data) is normalized to 1, so that cross-validation errors below 1 indicate that the model is explaining part of the variance of the held-out data. Figure 2A shows the average mean squared error across the cross-validation folds with the standard deviation over folds indicated with error bars. The minimal cross-validation error with standard PLS is obtained with four latent factors. Graph-regularized PLS appears to be more resistant to overfitting, with slightly lower cross-validation error at four latent factors and no substantial increase in error as the number of latent factors increases. Again, cross-validation error suggests that four latent factors should be used in the model. As a negative control, we randomly paired promoter sequences with expression profiles, so that we used real expression data and promoter sequences but lost the correspondence between sequence and expression, and we performed standard PLS and graph-regularized PLS . As can be seen from Figure 2A, both standard PLS and graph-regularized PLS on randomized data overfit with the very first latent factor, indicating that the performance obtained on the real data is meaningful.

### Latent factors map to germline-specific expression trajectories

**Figure 3:**
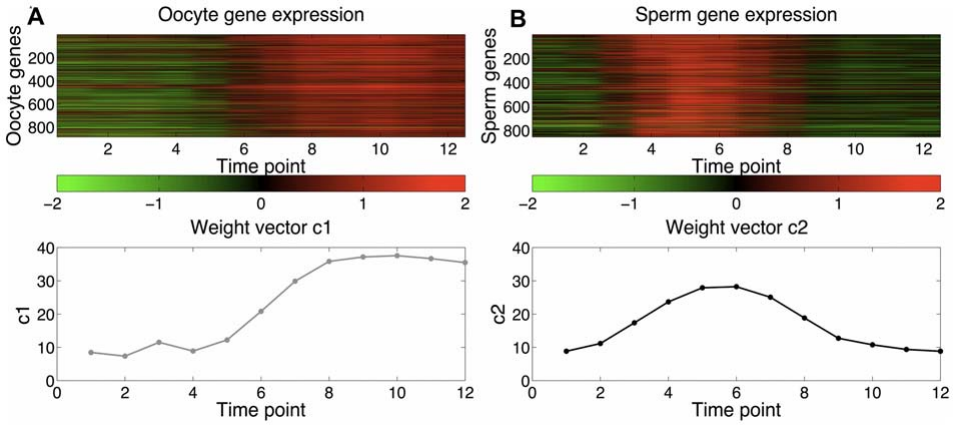
Correlation of germ cell expression patterns and PLS expression weight vectors. Oocyte and sperm gene expression patterns are strongly correlated with 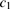 and 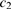, respectively. (A) Oocyte gene expression versus 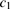. (B) Sperm gene expression versus 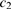.

By analyzing separate microarray expression data from germline mutants, the previous study also identified two gene sets consisting of sperm and oocyte genes [7], which we used in our analysis of the wild type developmental gene expression profiles. First, we estimated the prediction error on each gene set as shown in Figure 2B. Clearly, the first and second latent factors account for the largest loss reduction for oocyte and sperm genes, respectively. To show that the first two factors dominate these two gene sets, we first examined the expression profiles of the two gene sets. In PLS, each weight vector 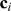 gives the weights over time points and can be interpreted as an expression pattern, and genes significantly influenced by the latent factor tend to follow this expression pattern. We plot the oocyte gene expression profiles together with 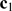 and sperm gene expression profiles with 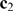 in Figure 3A and 3B. The gene expression profiles are strongly correlated with the corresponding weight vectors, indicating that the first two factors are able to retrieve the expression patterns of these two gene sets, respectively. Furthermore, we used functional enrichment analysis to confirm that the genes identified based on correlation with weight vector by these two factors are indeed enriched for oocyte and sperm genes, respectively (Figure S1(A,B)).

### Interpretation of motif weight vectors

**Figure 4:**
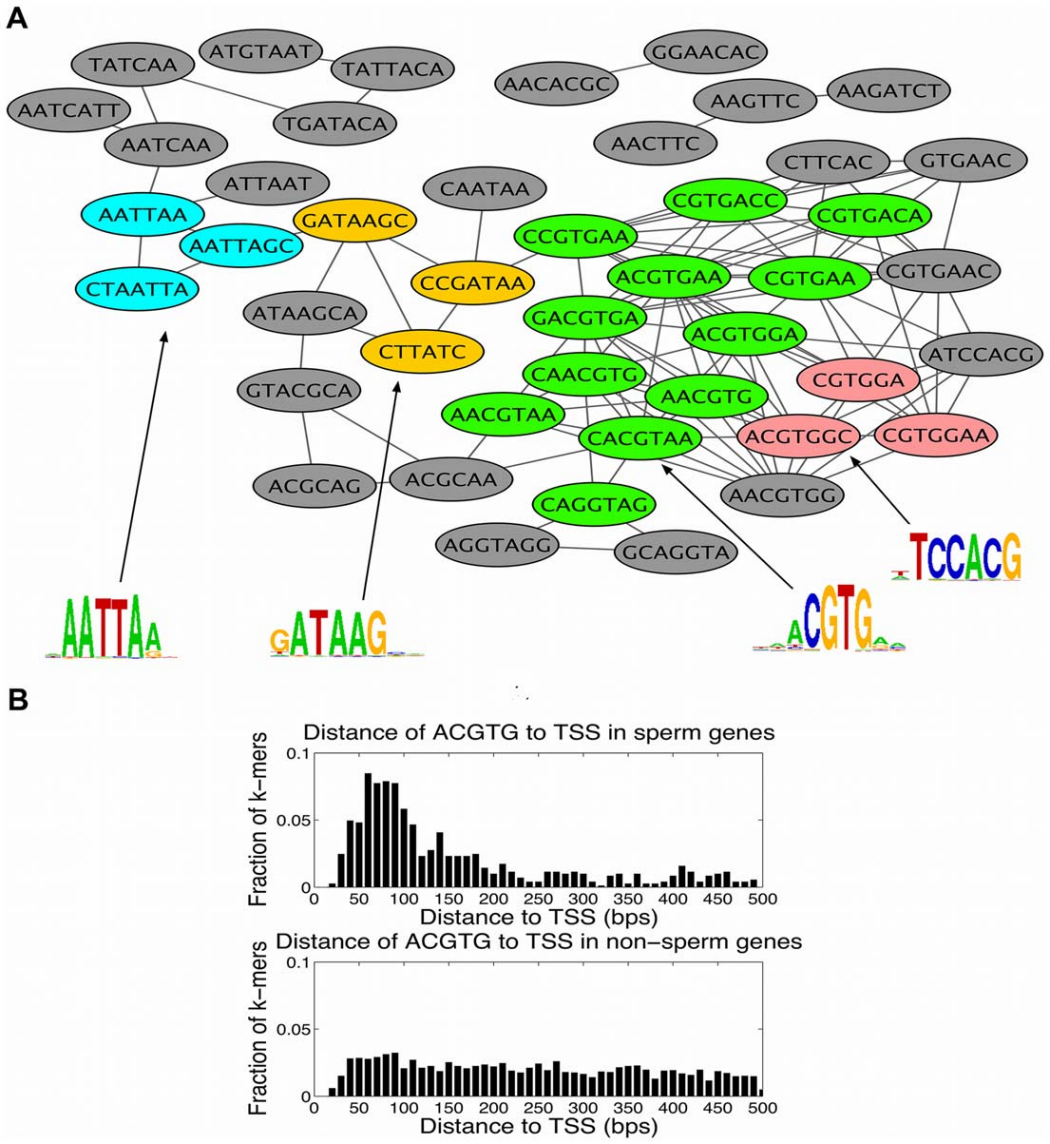
Sperm motifs determined by graph-mer analysis and positional bias of motif ACGTG. (A) Sperm motifs extracted from graph-mer output. The graph-mer consisting of the top 50 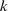-mers ranked by 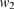. Graph motif patterns identified in the form of 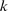-mer clusters using the MCODE plug-in [9] in Cytoscape are shown in different colors, with each subgraph summarized by a PSSM generated through hierarchical sequence agglomeration of the corresponding 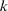-mers. Both the ELT-1 motif GATAA and the bHLH motif ACGTG are found in this way. (B) Distribution of distance of motif ACGTG to TSS (measured in base pairs) in sperm genes versus non-sperm genes. Motif ACGTG occurs more frequently within 200bp upstream of the TSS in sperm genes relative to non-sperm genes, giving us more confidence in its contribution to sperm gene expression.

**Figure 5:**
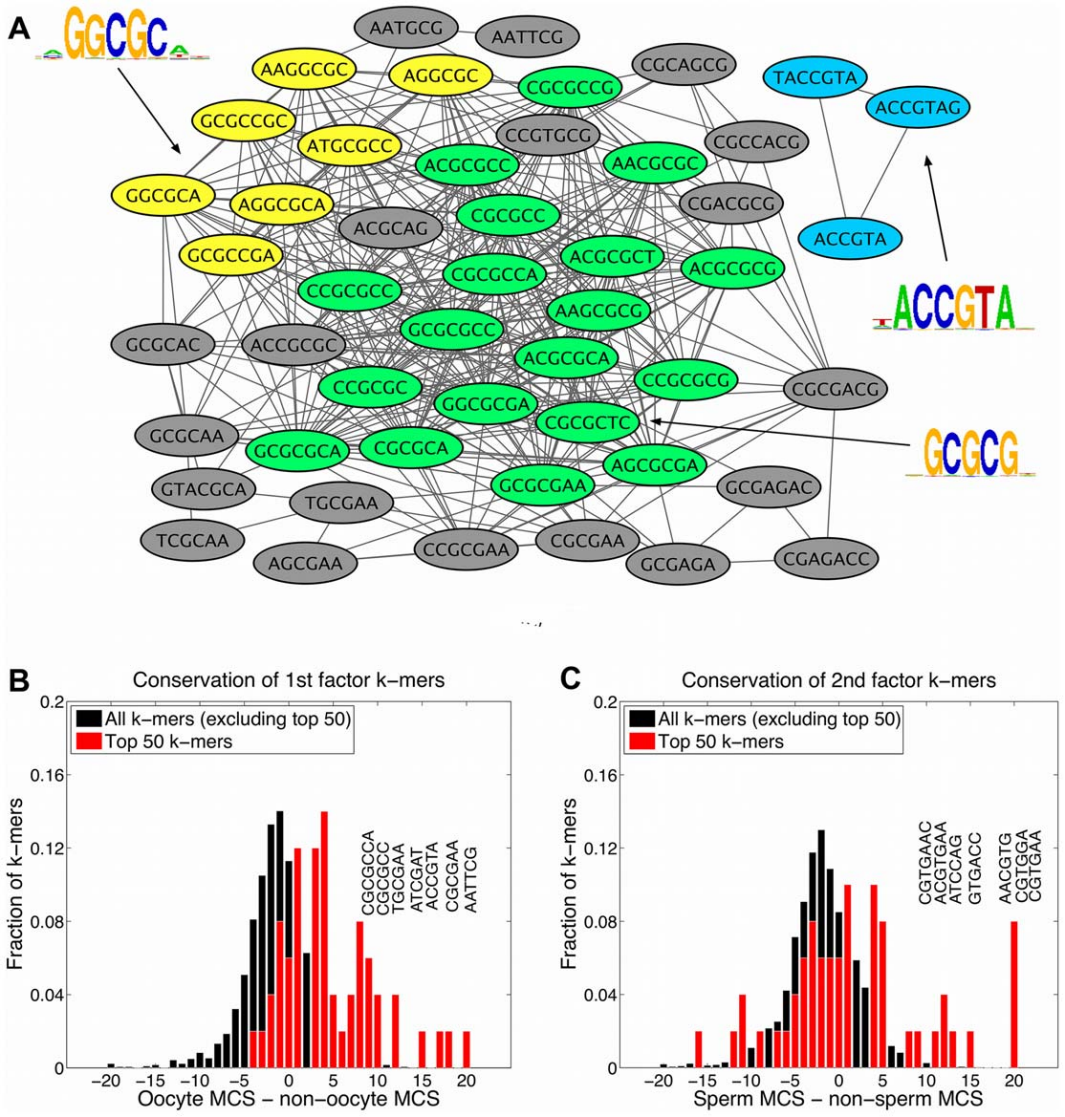
Oocyte motifs determined by graph-mer analysis and conservation of graph-mer derived oocyte and sperm motifs. (A) Top 50 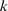-mers ranked by the weight vector 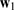, depicted as a graph-mer, which are associated by the PLS procedure to the expression pattern of oocyte genes. Graph motif patterns were identified in the form of 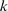-mer clusters using the MCODE plug-in in Cytoscape. PSSMs generated through hierarchical sequence agglomeration of the corresponding 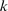-mer sets are indicated, revealing several CG-rich motifs. (B) Analysis of oocyte 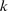-mer conservation using the motif conservation score (MCS). The plot shows the distribution of (oocyte MCS
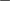
non-oocyte MCS) for top 50 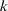-mers versus remaining 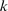-mers in 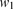. The score distribution for the top 50 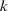-mers has a heavy right tail, showing that as a distribution, the top 50 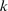-mers have higher oocyte-specific conservation scores as compared to other 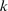-mers (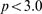
e-13 by a one-sided KS statistic). Significantly conserved 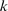-mers are annotated, including CG-rich 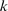-mers for oocyte genes. (C) Distribution of (sperm MCS
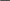
non-sperm MCS) for top 50 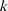-mers versus remaining 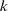-mers in 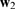. The score distribution for the top 50 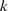-mers has a heavy right tail, showing that the top 50 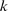-mers have higher distribution of sperm-spefic conservation scores than other 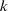-mers (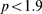
e-5, one-sided KS statistic). Significantly conserved 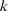-mers are annotated, including ACGTG motif for sperm genes.

In PLS, each weight vector 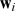 corresponds to a set of motifs (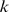-mers) that help to explain expression patterns in the direction 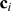. The 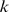-mers with largest coefficients in 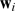 are the most important variables for predicting the projection of the expression patterns of genes onto 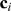. To identify motifs relevant for sperm and oocyte gene sets, we selected the top 50 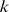-mers ranked by 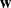 and examined the 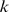-mer graphs corresponding to the first two latent factors. Clusters in the graph that are identified by MCODE [9] represent motif patterns and hierarchical sequence clustering is performed to generate corresponding PSSMs. Figures 4A and 5A show the graph-mer representation of the top 50 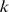-mers, motif patterns and PSSMs for the first two factors.

From the second factor, we successfully found the ELT-1 (‘erythrocyte-like transcription factor’) motif GATAA and bHLH (‘basic helix-loop-helix’) motif ACGTG, as shown in Figure 4A. The ELT-1 protein is a transcriptional activator that can recognize the GATA motif, is highly expressed in the germ line, and has as potential targets a number of genes encoding major sperm proteins [10]. The bHLH proteins act through E-box elements with consensus CANNTG; the canonical E-box is CACGTG. bHLH proteins have been found to act at the E-box and influence hormone-induced promoter activation in mammalian Sertoli cells, which are required to maintain the process of spermatogenesis [11]; however, this motif has not previously been associated with spermatogenesis in *C. elegans*.

For the first latent factor, the top ranked motifs are CG-rich sequences as shown in Figure 5A, which are highly enriched in oocyte gene promoters (Figure S2), suggesting a potential role in oogenesis or regulation of oocyte gene expression. We found further evidence supporting the functional relevance of learned motifs for the first two latent factors by performing gene set enrichment analysis, which showed that oocyte and sperm gene sets are enriched in the corresponding 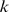-mer hits (Figure S1(C,D)).

### Positional bias and conservation of motifs

Since functional motifs sometimes exhibit a spatial bias in the promoter region – for example, overrepresentation close to the transcription start site (TSS) – we performed positional analysis of top ranked motifs by examining their distance to the TSS in sperm genes versus non-sperm genes. We observed that the sequence element ACGTG displayed strong positional bias towards the TSS of sperm genes. Figure 4B plots the distribution of distance of ACGTG to TSS in sperm genes versus non-sperm genes, showing that ACGTG is found far more frequently within 200bp upstream of the TSS of sperm genes but displays a fairly uniform distribution relative to TSS in non-sperm genes. This result indicates that motif ACGTG was significantly overrepresented immediately upstream of sperm genes, giving us additional confidence in the motif's contribution to sperm gene expression.

To look for evidence of the functional roles of CG-rich and other highly weighted motifs, we considered conservation patterns of these sequences. *Caenorhabditis briggsae* is closely related to *C. elegans* and is frequently used in comparative genomics studies in worm. One expects that motifs responsible for a biological function that is shared by the two species, such as oogenesis, would be under evolutionary pressure and therefore conserved in the promoter regions of orthologous genes contributing to this function. We studied the conservation of all 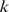-mers between the two species and found that highly ranked 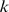-mers, where rankings are induced by the 1st and 2nd factor, tended to be more conserved in the oocyte genes and sperm genes, respectively. Specifically, we computed the motif conservation score (MCS) [12] of each 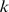-mer by comparing its conservation rate 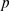 to its expected rate 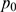, estimated using 500 random 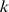-mers of the same length. A conserved occurrence of a 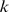-mer is an instance of the 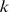-mer in the *C. elegans* genome, for which it is also present in the *C. briggsae* ortholog. We reported MCS as a Z-score (
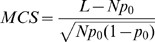
) measuring the significance of observing 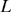 conserved occurrences out of total 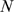 occurrences. To assess the significance of inferred 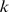-mers for oocyte and sperm gene sets, we focused on motif conservation in sperm and oocyte genes relative to non-sperm and non-oocyte genes. To do this, we computed the MCS of each 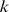-mer in both oocyte genes and non-oocyte genes, and we plotted the distribution of the difference of these two MCS scores for top 50 ranked 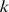-mers in the 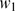 versus remaining 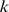-mers, as shown in Figure 5B, bottom panel; similarly, Figure 5C shows the difference of the MCS scores for sperm genes and non-sperm genes for the top 50 ranked 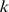-mers in 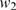 versus the remaining 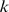-mers. For both oocyte and sperm gene sets, the score distribution for the top 50 k-mers has a heavy right tail relative to other 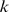-mers, showing that the top 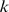-mers have higher oocyte- and sperm-specific conservation. To confirm the significance of this observation, we performed a one-sided Kolmogorov-Smirnov (KS) test and found that the rightward shift was highly significant in both cases (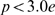-13 and 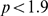
e-5 for oocyte and sperm 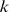-mers, respectively). The 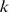-mers that are most significantly conserved in oocyte and sperm genes, relative to non-oocyte and non-sperm genes, are also annotated in Figure 5B and 5C; these include the ACGTG motif for sperm genes and CG-rich 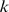-mers for oocyte genes.

### Targets of CG-rich motifs are expressed in the germline

Relatively little is known about transcriptional regulation of oocyte genes. To gain additional evidence supporting a functional role for learned motifs, we examined the *in situ* expression patterns of genes enriched with those motifs. We searched for a subset of EST (expressed sequence tag) clones known as YK clones of each gene in WormBase (http://www.wormbase.org) and looked at *in situ* expression patterns at the L4-adult stage associated with each YK clone in the Nematode Expression Pattern Database (NEXTDB http://nematode.lab.nig.ac.jp/db2/index.php).

The *in situ* analysis provides direct evidence about where the genes are expressed, and we expect that genes highly ranked by motif hits are more likely to be germline expressed. To obtain a ranked gene list for each of the three motifs in Figure 5A, we first defined the gene group associated with the first factor based on 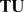 values (see Methods). For each motif, we ranked genes within the gene group by counts of 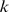-mers of that motif and came up with a list consisting of top 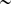
80 genes. Table 1 summarizes the *in situ* expression patterns of genes associated with motif 1 (GGCGC), motif 2 (GCGCG) and motif 3 (ACCGTA). We split each gene list into two groups, those already known to be oocyte genes, and genes with high motif scores not already defined as oocyte genes. For each group, Table 1 shows number of genes examined; the number of genes with an *in situ* pattern; and percentage of genes expressed in germline tissues only, in both germline and somatic tissues, and somatic tissues only.

**Table 1:** *In situ* analysis of genes enriched with CG-rich motifs.

Motif	Previously identified as oocyte genes	# genes	# genes with *in situ* pattern	% Germline only	% Germline & somatic	% Somatic only
Motif 1	yes	29	28	71%	7%	5%
(GGCGC)	no	52	37	73%	8%	13%
Motif 2	yes	31	25	80%	4%	4%
(GCGCG)	no	55	43	74%	14%	5%
Motif 3	yes	26	16	94%	0%	0%
(ACCGTA)	no	62	38	76%	10%	0%

For each graph-mer derived motif, we identified the set of genes associated to the motif based on latent factor analysis (see Methods). Each gene list was further split into two sets: genes that had been previously identified as oocyte genes based on mutant expression data and those not identified as oocyte genes by this previous analysis. The table shows the number of genes associated to the motif; the number of genes having an *in situ* pattern in the NEXTDB database; and genes expressed in germline tissues only, in both germline and somatic tissues, and somatic tissues only as a percentage of genes with an *in situ* pattern. The results show that even among genes not previously identified as oocyte genes, more than 70% of genes examined were dominantly expressed in germline tissues rather than somatic tissues. This percentage is much higher than seen overall for genes that were not previously called oocyte or sperm without considering motif information (20%), suggesting a functional role of CG-rich motifs in germline expression.

Over all three motifs, 7% of the genes have detectable *in situ* staining. Of those, an average of 78% stain only in the germ line, and with more than 80% of genes previously identified as oocyte genes staining in the germ line.

More than 70% of genes that had not previously been identified as oocyte genes (based on mutant expression profiling) were also dominantly expressed in germline tissues rather than somatic tissues. In the study that defined the oocyte and sperm gene sets [7], about 20% of genes that were not identified as oocyte or sperm had the germline expression by *in situ* analysis. Table 1 shows that for the genes that were associated with oocyte motifs 1, 2 and 3 via latent factor analysis – but had not previously been identified as oocyte genes – 37/52, 43/55, and 38/62 showed germline expression. All these proportions are very significantly higher than the background percentage of 20% (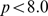
e-16 for all motifs by a proportions test). These results provide additional evidence that we are learning functional motifs that contribute to germline expression.

### Comparison with principal component analysis

Principal component analysis (PCA) is a widely used dimensionality reduction technique that extracts from the data matrix a sequence of orthogonal vectors, or principal components, that capture the directions of maximal variance in the input data. PCA is frequently used on either rows (genes) or columns (experiments) of a gene expression matrix for visualization or preprocessing prior to other kinds of analysis [13]. By contrast, PLS is a supervised method that, in our context, determines weight vectors 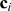 as directions in gene expression space having maximal covariance with latent factors in motif space. Both PCA components and PLS weight vectors are interpreted as gene expression patterns. However, principal components are learned from gene expression data only, while weight vectors 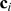 are found based on a linear mapping from motif space to gene expression space.

**Figure 6:**
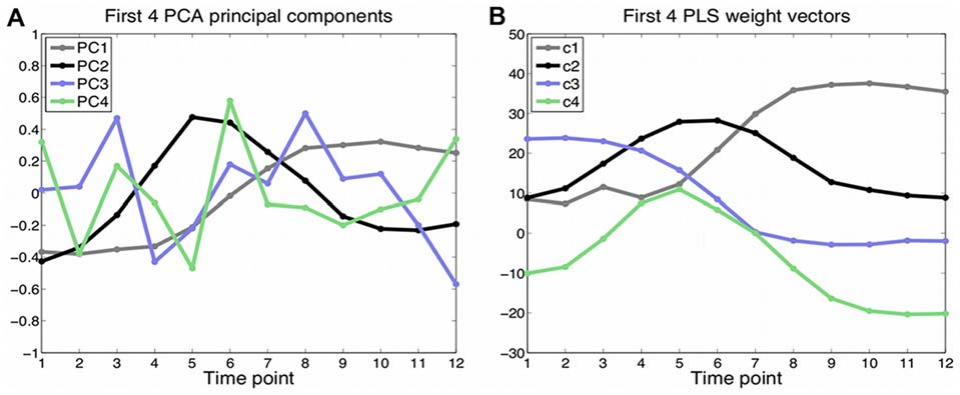
Comparison of PCA components and PLS expression weight vectors in gene expression space. The first and second principal components bear some similarity to corresponding PLS weight vectors 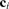, 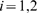, but all principal components are less smooth than in PLS. (A) PCA identifies the first four directions (PC_1_, PC_2_, PC_3_ and PC_4_) that have maximal variance in gene expression space. Principal components are plotted v.s. time. (B) Graph-regularized PLS learns weight vectors (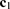, 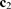, 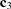 and 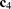) based on a linear mapping from motif space to gene expression space. Weight vectors are plotted vs. time.

We were interested in comparing our (graph-regularized) PLS results with standard PCA in order to assess the value added by the motif information and supervised learning formulation. We anticipated some concordance of results, since directions that capture little variance in the expression data will also fail to have significant covariance with motif latent factors. Figure 6A and 6B plot the first four PCA components versus PLS weight vectors. The first and second PCA components indeed bear some similarity to the first and second PLS weight vectors and to some extent resemble the oocyte and sperm gene expression patterns, respectively. Since these two gene sets are fairly large and follow distinct expression patterns, they account for a significant portion of gene expression variance, and so it is not surprising that the first PCs show correlation with these patterns. However, all the principal components are less smooth, as expression trajectories, than their corresponding PLS weight vectors, and the smoothness of the PCs deteriorates more rapidly than in PLS as the number of principal components/latent factors increases. It therefore appears that PLS uses motif information to provide some degree of regularization on the weight vectors, leading to smoother expression patterns corresponding to latent factors.

To confirm that the PLS-derived motifs could not be determined from analysis of the first and second principal components (PC_1_ and PC_2_), we performed the following motif discovery procedure: we identified the sets of genes that are highly correlated with PC_1_ and PC_2_, and ran the AlignACE motif discovery program on the promoters of these genes, yielding 58 and 89 motifs, respectively (see Text S1). In both cases, the top-ranked motifs were dominated by AA-rich and GG-rich motifs that likely come from low complexity regions (Figure S5). A few CG-rich motifs appear in the AlignACE list for PC_1_, but with relatively low MAP scores; only one motif from the list for PC_2_ matches any of the PLS-derived sperm motifs, and it occurs low in the ranking (rank = 33) with relatively weak MAP score. We conclude that analysis of the principle components does not retrieve the full motif information discovered by the PLS latent factors. This result underscores the importance of our predictive framework, mapping sequence to expression, rather than relying on correlation with expression and performing motif analysis after the fact.

Since the third and fourth PLS latent factors represent much smoother and quite different expression patterns than their PCS counterparts, we examined whether the genes associated to these factors based on motif and expression similarity (see Methods) may have common functions. While there were few genes associated to the fourth PLS factor (18 genes) showed no enrichment for GO terms, the gene set for the third PLS factor was significantly enriched for 54 GO terms (using a threshold of 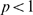
e-4, uncorrected hypergeometric people), of which the majority involved metabolism (32/54) and almost half of these were specific to amino acid metabolism (15/54). These genes are not enriched for germline expression, suggesting that our analysis has uncovered an independent co-regulation of a set of gene functions that might have been swamped out by the stronger germline information using other techniques.

### Comparison with clustering

Finally, we compared our results with standard cluster-first analysis, using hierarchical clustering to identify 5 distinct gene clusters and applying the AlignACE motif discovery program to the promoters of each cluster in order to find over-represented motifs (Text S1). We identified two clusters (Clusters 1, 2) with subtly different expression patterns both resembling the expression signature of oocyte genes and one cluster (Cluster 3) similar to the sperm gene expression signature (Figure S6(A,B,C)). AlignACE returned lists of 47, 53 and 36 motifs for these three clusters, and as in the principal component analysis, the top ranked motifs in all cases were dominated by low-complexity AA-rich and GG-rich motifs (Figure S6(D,E,F)). A handful of low-ranked motifs with relatively poor MAP scores for Clusters 1 and 2 resembled two of the CG-rich 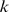-mers identified through the first PLS latent factor; for Cluster 3, none of the AlignACE motifs were similar to the sperm-specific 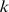-mers identified by the second PLS latent factor (Text S1). We conclude first that PLS avoids many presumably spurious motifs from low complexity regions while finding true germline-specific motifs that are missed through standard cluster-based analysis.

## Discussion

There have still been relatively few methods that integrate mRNA expression and promoter sequence data beyond “cluster-first” motif discovery. Beer and Tavazoie [14] similarly sought to reverse the information flow implied by clustering, to see how well motif content could predict expression patterns; in their case, however, expression patterns were identified with static clusters, motifs were discovered based on these clusters, and the learning task was the prediction of cluster membership rather than vector-valued expression profile. Ernst et al. [15] proposed a time-ordered hierarchical model for integrating motif and time series expression data, where motifs were associated with up/down bifurcations of expression profiles at particular time points; this method used static motif data rather than learning motifs. Segal et al. [16] combined promoter sequence and expression data within a probabilistic relational models framework to learn “modules” supported by both data sources; rather than learning motifs de novo, the algorithm was seeded with database motifs which could then be refined during expectation-maximization iterations. In our own previous work on the MEDUSA algorithm [17], we discretized expression data and used a boosting-based algorithm to discover motifs and assemble a regulatory program that predicts up/down expression of target genes. MEDUSA is well-suited to perturbation experiments and performs well even for small perturbation data sets [18]. In the current setting, where expression levels in consecutive time points are highly correlated and expression trajectories are smooth over time, discretizing the expression levels incurs a significant loss of signal, which we avoid by moving to a regression framework.

There have been several other regression based motif discovery approaches related to our work. For example, REDUCE [19] was the original method to use correlation between 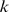-mers and differential expression for motif discovery. REDUCE, however, uses each experiment independently, where we use multivariate PLS to treat full expression trajectories as the output space. To weight the benefits of regression with a multivariate output, we also tried fitting a separate graph-regularized univariate PLS model on each time point separately. We found that multivariate PLS outperforms univariate PLS (Figure S3), suggesting that correlating motifs with full expression patterns is more statistically accurate than performing regression one experiment at a time, at least in the case of correlated experiments such as time series data. Moreover, there was substantial overlap in the motif information inferred from nearby time points (see Text S1), showing that fitting a separate model for each time point entails a good deal of redundancy.

More recently, Zhang et al. [20] used PCA to define a basis of univariate response variables in the output space and then performed a REDUCE-like regression onto each variable to collect a set of motifs. In our work, by doing multivariate regression, we retain more structure in the solution, for example, a stratification of the output space by images of latent factors, each one corresponding to a characteristic time expression profile. We also note that lasso regression has been used elsewhere for learning regulatory networks in bacteria using time course expression data [21], and standard PLS has been used with a collection of known motifs in linear modeling of expression data in yeast and bacteria [22]. Finally, graph-based motif representations have been used previously by other groups, for example Naughton et al. [23], but this work again falls into the “cluster-first” category in that it seeks to find overrepresented motifs for a predefined gene set. By contrast, we learn motifs via a global regression problem, and the graph structure is encoded as a constraint on the solution.

A number of recent studies have expanded beyond the linear regression framework by introducing various kinds of non-linearity. First, various authors have extended standard linear models by proposing that certain sets of motifs have synergistic effects. For example, a synergistic pair of TFs can be modeled by including a term in the regression model for each of the individual motif counts as well as a third term for the product of the counts, as recently reviewed [24]. However, introducing too many of these additional non-linear terms greatly increases the risk of overfitting; for a typical pair of TFs, the count of co-occurrences is simply too sparse to estimate the synergistic parameter. These models require careful feature selection strategies; moreover, they mostly assume that the motifs are known and fixed, whereas we are performing *de novo* motif discovery. Second, motivated by biochemical models, several studies propose that the relationship between motif counts or TF occupancy scores (in the case of PSSMs) and log expression change is not linear and make use of a non-linear transfer function. Recent work using a probabilistic framework to predict the 1D anterior-posterior positioning of expression “stripes” in the early Drosophila embryo from *cis* regulatory module (CRM) sequences can be seen as an elegant example of this idea [25]. In this case, a logistic transfer function converts occupancy scores, computed from the space of configurations of TFs in the CRM, into sharp stripe boundaries. In our setting, however, we are learning from microarray expression data, which gives average (and noisy) measurements over a large population of cells with large underlying variation of expression levels. It is unclear whether mRNA expression data allows us to observe and model biochemically-expected non-linearity in this situation. Third, when confronted with a multi-variate response, such as in time series expression profiles, some authors have used a model where each motif count/occupancy score contributes linearly to the expression pattern at each time point (as we do) but the time points are connected by use of non-linear basis functions such as splines [26]. However, we find that the smoothness of the PLS-derived expression patterns comes for free as a result of the regularization choices in our method, so in our hands the smoothness prior did not seem to be statistically necessary.

Finally, our method can be applied to even more sparsely sampled time series covering a broader range of developmental stages. As a proof-of-principle, we applied graph-regularized PLS to a full life cycle *C. elegans* developmental time course consisting whole-animal gene expression profiles from egg to adult [27] (see Text S1). In this setting, the first latent factor contained germline-specific motifs similar to the ones found in the analysis of our main data set, while the next second and third latent factors were associated with more diverse biological functions (Figure S4). These results suggest that our approach can discover the structure of gene regulatory programs, in the form of latent factors corresponding to sequence patterns and expression trajectories, at a range of developmental time scales.

## Materials and Methods

### Standard partial least squares regression

Since our algorithm builds on ideas from PLS regression, we first describe how to use standard PLS to iteratively learn a linear mapping from the promoter sequences of genes, as represented by their 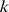-mer counts, and their mRNA expression profiles. Formally, using a training set of 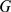 genes, PLS takes a motif matrix **X** (dimension 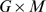, where 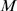 is the number of 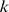-mers), representing the individual 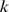-mer counts for each gene, and a gene expression matrix by **Y** (dimension 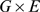, where 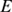 is the number of experiments). Here, the columns of **X** represent the independent variables (features) and the columns of **Y** are the response variables; we also call **X** the input matrix and **Y** the output matrix. PLS then performs the following steps:

- Scale **X** and **Y** so that each column of the input and output matrices has zero mean and unit variance.
- Perform dimensionality reduction by construction of latent factors 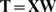: Construct 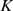 *weight* vectors, placed as column vectors in **W** (dimension 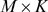), and corresponding *latent* factors, placed as column vectors in **T** (dimension 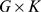), where the weight vectors are chosen so that the latent factors have maximal covariance with directions in the multivariate response **Y**.
- Use the latent factors **T** to predict **Y**: Regress **Y** against the latent factors using ordinary least squares (or ridge) regression,
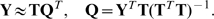

- Obtain the matrix **B** of regression coefficients:
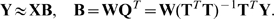

We split genes into test and training sets for cross validation experiments. Training data including motif matrix **X** and gene expression matrix **Y** were used to learn matrix of regression coefficients **B**. And we assessed predictive power of PLS on test data 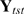 and 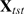 by normalized mean squared error (NMSE):
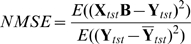
(1)where 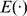 denotes the expected value and 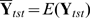.

PLS not only provides a solution to the regression problem, but it also describes the covariance structure between **X** and **Y**. It constructs 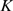 weight vectors 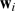 in the input space 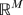 and corresponding vectors 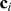 in the output space 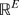, where 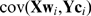 is maximal. Intuitively, each weight vector 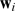 corresponds to a set of motifs (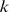-mers) that helps explain expression patterns in the direction 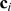. The 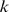-mers with largest coefficients in 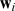 are the most important variables for predicting the projection of the expression patterns of genes onto 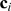.

### SIMPLS algorithm

There are a number of variants of PLS, each of which defines and solves an optimization problem for constructing the weight matrix **W**. We use the SIMPLS (Statistically Inspired Modification of PLS) algorithm [28], which optimizes an objective function defined on the matrix 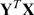. The latent factors 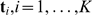 in T are sequentially built by estimating weight vectors 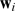 as follows:

For 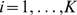:

- Maximize the covariance between 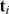 and **Y**:
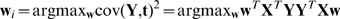
(2)where 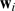 is a unit vector.
- Impose orthogonality constraints 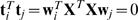 for all 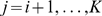, by deflating 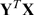:
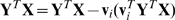
(3)where (i) If 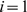, 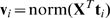.

(ii) If 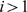, 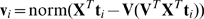 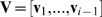.

### Regularized partial least squares regression

We now modify the PLS algorithm with the dual goals of (1) making the solution more interpretable and (2) regularizing the optimization problem, to reduce overfitting. We impose two constraints to achieve these goals. First, we use a lasso (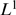) constraint [2] to promote sparsity in the weight vectors 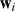, that is, drive the weights for many 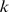-mers to zero. Sparsity is clearly attractive since fewer 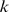-mers contribute to the solution, making it easier to identify the most important motifs. The lasso constraint over coordinates 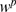 of weight vector 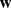 takes the form:
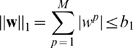
(4)


For the second constraint, we want sequence-similar 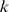-mers to have similar coefficients in the weight vectors, so that a group of similar 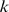-mers are more likely to act as a single motif pattern in the regression problem. We define a graph structure on the 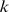-mers where we place an edge 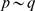 if the Hamming distance between the pair of 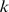-mers 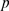 and 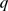 is less than threshold 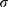. Since 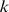-mers represent potential binding sites in double-stranded DNA, here we take the distance between two 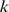-mers 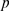 and 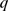 to be the minimum of the Hamming distances 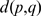 and 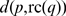, where 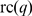 is the reverse complement of 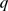. We then impose a smoothness constraint in the form of the graph Laplacian [29], as described below. The Laplacian matrix 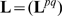 for an unweighted graph is defined as
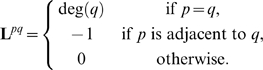
(5)where 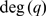 denotes the degree of 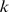-mer 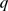, the number of edges that connect 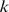-mer 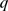 with other 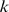-mers. If we write 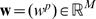 as a column vector and view it as a function on the graph – i.e. a function that assigns a weight 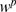 to each vertex 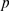 – then we can use the graph Laplacian to compute a quadratic form on 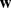 that satisfies the relationship [30]:
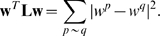
(6)Equation (6) shows that this quadratic form measures the *smoothness* of 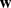 with respect to the graph: the quadratic form is small when the function's values vary smoothly over adjacent nodes, so that the weights for sequence-similar 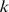-mers are close in value. Therefore, the second constraint that we impose is precisely on the size of the quadratic form, enforcing smoothness on the weight vector 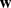:
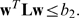
(7)


**Figure 7:**
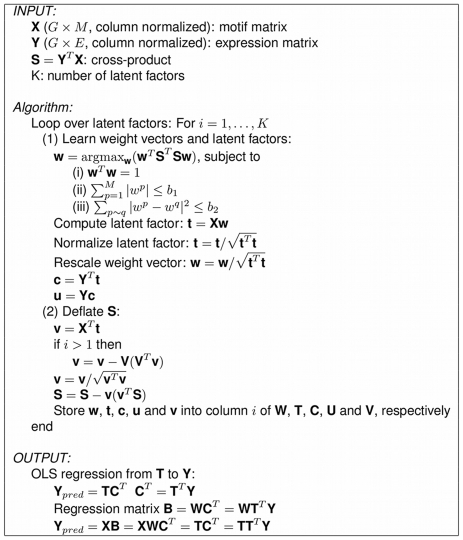
Pseudocode for graph-regularized PLS. A pseudocode description of the iterative PLS procedure, enforcing sparsity and Laplacian constraints on motif weight vectors.

A pseudocode description of the graph-regularized PLS algorithm is given in Figure 7.

### Filtering *k*-mer features


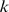
-mer features with very sparse genome-wide counts are unlikely to improve the loss function – since they only only in a handful of promoters – and can contribute to overfitting. In order to eliminate 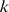-mers with infrequent counts prior to training, we filtered the 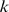-mer feature set based on expected counts on background sequences. We constructed the background sequences by shuffling exon sequences 100 times and ranked 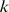-mers by the 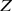-score [31]: 
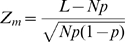
, where 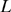 is the number of the 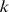-mer in all promoter sequences, N is the length of all shuffled exon sequences, and 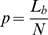 is number of the 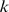-mer in all shuffled exon sequences divided by N. (Note that shuffled intergenic sequences could also be used to generate the random model.) We kept the top 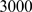 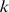-mers and built the motif matrix containing counts of 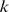-mers in promoter sequences. We found that this filtering step significantly improved cross-validation performance.

### Hierarchical sequence agglomeration

For each latent factor 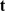, we rank 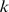-mers by their components in the corresponding weight vector 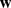 and perform motif analysis on the top 50 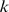-mers. Those 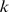-mers are first displayed in the form of a motif graph via Cytoscape [32], in which an edge between two 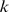-mer nodes indicates similarity. We used the MCODE Cytoscape Plugin [9] to find 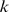-mer clusters (highly interconnected sets of sequence-similar 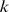-mers) in the graph. Each 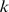-mer cluster represents a motif pattern consisting of slightly different 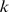-mers.

Finally we perform a hierarchical sequence agglomeration algorithm to generate position-specific scoring matrices (PSSMs) for 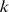-mer clusters. Within each 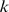-mer cluster, each 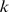-mer is treated as a seed PSSM (using background nucleotide probabilities for smoothing), and then the algorithm iteratively merges similar PSSMs until a single PSSM is learned as the binding site model.

A position-specific scoring matrix (PSSM) is represented by a probability distribution 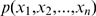 over sequences 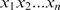, where 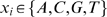. The emission probabilities are assumed to be independent at every position such that 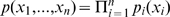.

When comparing two PSSMs 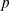 and 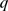, we allow offsets between their starting positions. We pad either the left or right ends with the background distribution and then define a distance measure 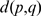 as the minimum over all possible position offsets of the JS entropy.
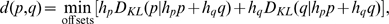
(8)where 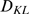 is the Kullback-Leibler divergence. Given that the position-specific probabilities are independent, one can easily show that 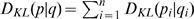. The relative weights of the two PSSMs, 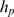 and 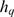, are here defined as 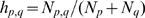, where 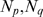 are the numbers of target genes for the given PSSM. The initial PSSMs are 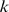-mers and the number of target genes are the number of promoter sequences with the 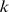-mer occurrence. The number of target genes for the newly merged PSSM will be the number of target genes combined for the two old PSSMs.

### Assigning genes to latent factors

To extract biological information from the algorithm output, we analyzed latent factors for potential gene groups and corresponding biological functions. To do that, we assigned each gene 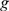 to the gene group associated with a factor 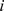 based on 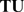 values. Here, the matrix 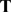 (respectively, 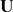) is formed by placing vectors 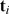 (respectively, 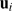) for latent factors 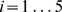 as column vectors (Figure 1). The value 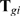 indicates how well 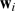 captures the 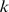-mer profile of gene 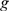, and the value 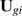 measures the similarity between 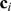 and expression profile of gene 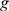. In contrast to traditional clustering, which only relies on gene expression to group genes, we integrate both sequence and gene expression information in learning potentially functional gene sets. For each gene 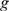, we computed 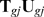 across all factors and chose factor 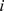 with the maximum value:
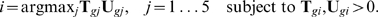



Since we suspected that only large 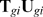 values indicated strong association of a gene 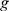 with factor 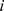, we assigned gene 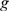 to factor 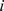 only when 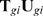 was in the top 20% of all 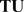 values. Although we use 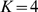 latent factors in our model, here we compute the representation with five factors, reasoning that if a gene is assigned to the 5th factor, it should not be included in our main analysis.

## Supporting Information

Figure S1 Correspondence between first and second latent factors and sperm and oocyte genes. (A,B) The set of all genes is split into oocyte and non-oocyte genes, or sperm and non-sperm genes, and the empirical cumulative distribution of correlation with c_i_, i = 1,2 is plotted. Oocyte and sperm genes are enriched towards the top of the gene expression correlation distribution. (C,D) The set of all genes is split into oocyte and non-oocyte genes, or sperm and non-sperm genes, and the corresponding empirical cumulative distributions of hits of top 50 *k*-mers in w*i*, i = 1,2 are plotted. Oocyte and sperm genes are enriched in *k*-mer hits corresponding to the 1st and 2nd weight vectors.
(3.03 MB TIF)

Figure S2 Correlation of weights with significance of enrichment in oocyte and sperm genes for the *k*-mers from 1st and 2nd graph-mer respectively. We plot the weights of *k*-mers in the first motif weight vector versus the −log_10_(p-value) for the enrichment of these *k*-mers in oocyte and sperm genes, as computed by the hypergeometric distribution. (A) For oocyte genes, −log_10_(p-value) is moderately correlated with w_1_ (Pearson coefficient = 0.65), and *k*-mers highly ranked by w_1_ had p-values between 10^−16^ and 10^−4^. This enrichment supports the functional relevance of PLS-derived *k*-mers from the first factor in oocyte genes. (B) For sperm genes, −log_10_(p-value) is somewhat correlated with w_2_ (Pearson coefficient = 0.35), though the correlation is weaker than that of oocyte genes.
(0.42 MB TIF)

Figure S3 Normalized mean squared prediction error on cross-validation test data. (A) Normalized mean squared error versus number of PLS iterations for standard univariate and multivariate PLS. At each iteration, standard univariate PLS learns twelve latent factors, corresponding to the twelve individual time points, while multivariate PLS learns one latent factor for all time points. Univariate PLS yielded a slightly lower test error than that of standard multivariate PLS after the 1st iteration; however, after one iteration, the univariate PLS corresponds to a collection of motif sets, each predicting a single experiment's gene expression changes, while multivariate PLS uses a single motif set to predict full gene expression trajectories. (B) Normalized mean squared error on test data by time point after the 1st univariate PLS iteration. Normalized mean squared error versus time point on all genes, oocyte and sperm gene sets. Univariate PLS reaches lowest prediction error on oocyte gene set at late time points when oocyte gene expression peaks. Similarly, prediction error on sperm gene set is small at middle time points when sperm gene expression peaks. Each time-specific univariate PLS models the motif-expression correspondence for the gene set differentially expressed at the given time point.
(0.40 MB TIF)

Figure S4 Latent factor analysis reveals graph-mers, expression patterns and significant associations of gene annotations. For each latent factor (i = 1…3), an associated mini graph-mer, extracted motif patterns and gene group are shown; annotations that are significantly enriched in each gene group are listed at the right (p<.001, uncorrected hypergeometric p-value), with p-values and number of genes associated with each annotation.
(2.02 MB TIF)

Figure S5 Motifs found by AlignACE in genes correlated with PC_1_ and PC_2_. (A) Top 40 AlignACE motifs in genes correlated with PC_1_ sorted by MAP score. Top ranked AA-rich and GG-rich motifs may result from low complexity regions, and several PCA motifs with relatively low MAP scores (e.g. MAP = 147.05, 90.77, 80.93) are similar to PLS 1st factor motifs. (B) Top 40 AlignACE motifs in genes correlated with PC_2_. Only one motif (MAP score = 101.03) is similar to our PLS sperm gene motif ACGTG from 2nd weight vector. None of the other PCA motifs matched any of the PLS 2nd factor motifs.
(7.58 MB TIF)

Figure S6 Motifs found by AlignACE in different gene clusters. (A) Expression patterns of genes in Cluster 1. (B) Expression patterns of genes in Cluster 2. (C) Expression patterns of genes in Cluster 3. (D) Top 40 AlignACE motifs found in Cluster 1 genes. (E) Top 40 AlignACE motifs found in Cluster 2 genes. (F) All 35 AlignACE motifs found in Cluster 3 genes.
(10.87 MB TIF)

Text S1 Supplementary results
(0.08 MB PDF)
