# Supplementary figures and images for: Learning “graph-mer” Motifs that Predict Gene Expression Trajectories in Development
Source: PLoS Comput Biol. 2010 Apr 29;6(4):e1000761. doi: 10.1371/journal.pcbi.1000761 (PMC2861633; doi:10.1371/journal.pcbi.1000761)

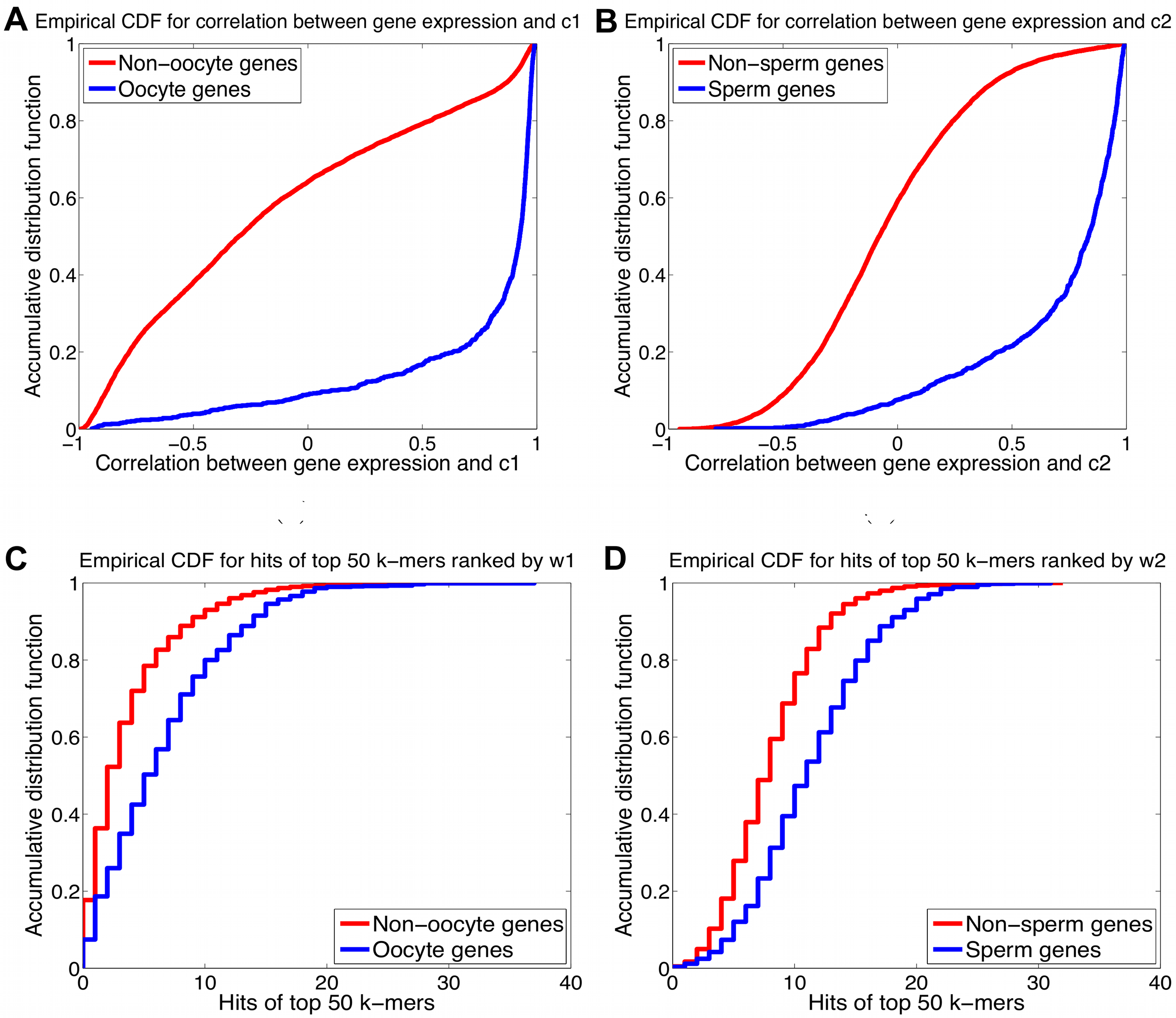

Supplement: Figure S1 — Correspondence between first and second latent factors and sperm and oocyte genes. (A,B) The set of all genes is split into oocyte and non-oocyte genes, or sperm and non-sperm genes, and the empirical cumulative distribution of correlation with ci, i = 1,2 is plotted. Oocyte and sperm genes are enriched towards the top of the gene expression correlation distribution. (C,D) The set of all genes is split into oocyte and non-oocyte genes, or sperm and non-sperm genes, and the corresponding empirical cumulative distributions of hits of top 50 k-mers in wi, i = 1,2 are plotted. Oocyte and sperm genes are enriched in k-mer hits corresponding to the 1st and 2nd weight vectors. (3.03 MB TIF) [file pcbi.1000761.s001.tif]

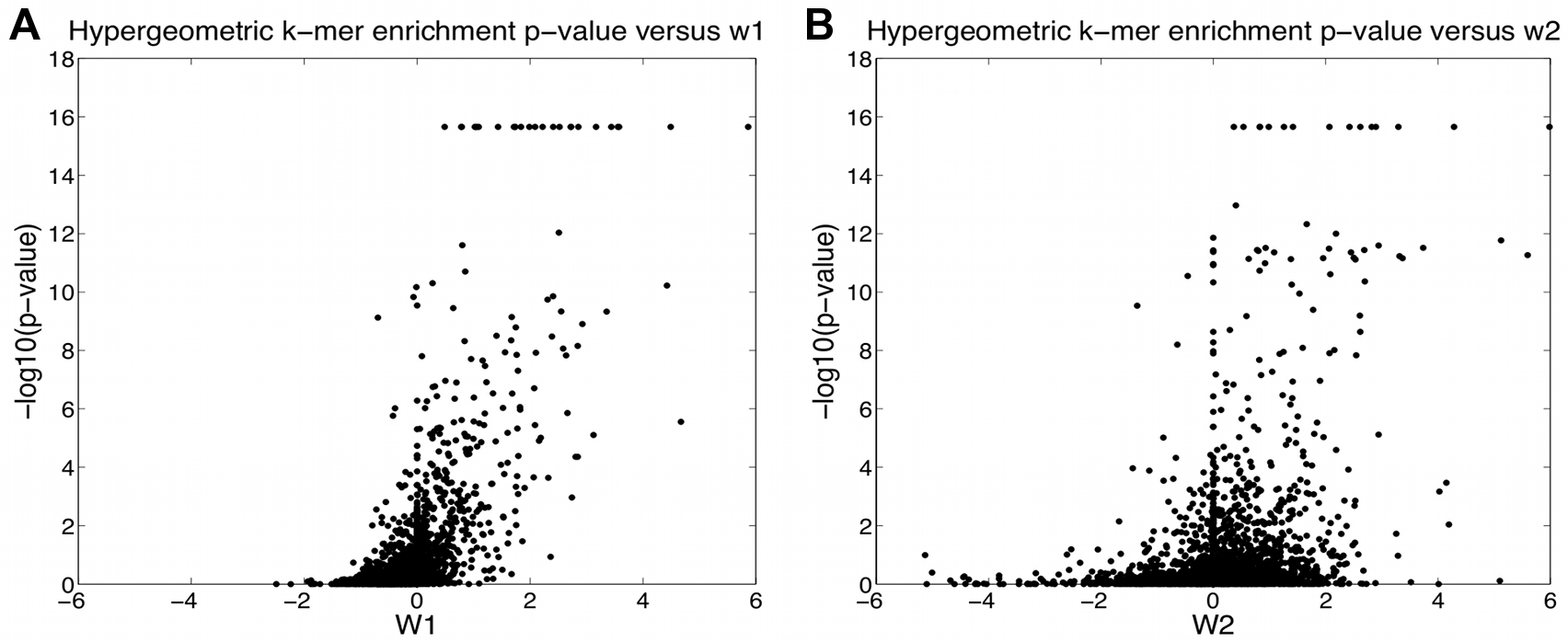

Supplement: Figure S2 — Correlation of weights with significance of enrichment in oocyte and sperm genes for the k-mers from 1st and 2nd graph-mer respectively. We plot the weights of k-mers in the first motif weight vector versus the −log10(p-value) for the enrichment of these k-mers in oocyte and sperm genes, as computed by the hypergeometric distribution. (A) For oocyte genes, −log10(p-value) is moderately correlated with w1 (Pearson coefficient = 0.65), and k-mers highly ranked by w1 had p-values between 10−16 and 10−4. This enrichment supports the functional relevance of PLS-derived k-mers from the first factor in oocyte genes. (B) For sperm genes, −log10(p-value) is somewhat correlated with w2 (Pearson coefficient = 0.35), though the correlation is weaker than that of oocyte genes. (0.42 MB TIF) [file pcbi.1000761.s002.tif]

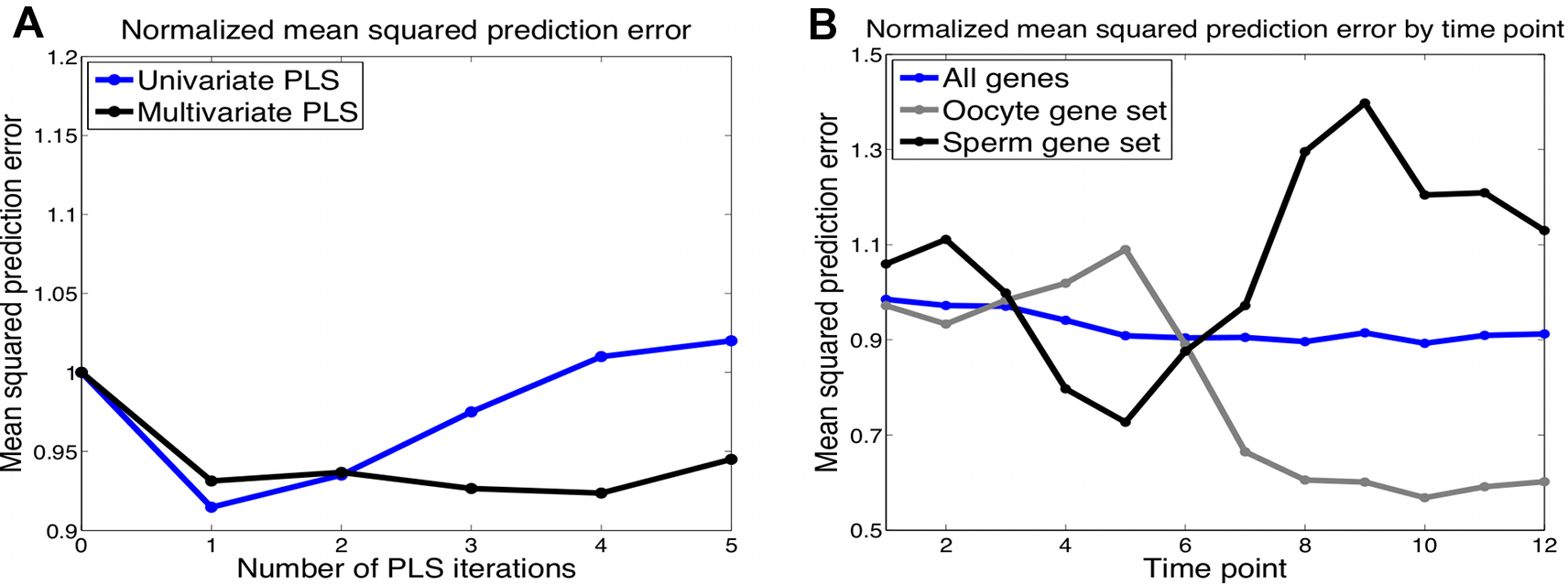

Supplement: Figure S3 — Normalized mean squared prediction error on cross-validation test data. (A) Normalized mean squared error versus number of PLS iterations for standard univariate and multivariate PLS. At each iteration, standard univariate PLS learns twelve latent factors, corresponding to the twelve individual time points, while multivariate PLS learns one latent factor for all time points. Univariate PLS yielded a slightly lower test error than that of standard multivariate PLS after the 1st iteration; however, after one iteration, the univariate PLS corresponds to a collection of motif sets, each predicting a single experiment's gene expression changes, while multivariate PLS uses a single motif set to predict full gene expression trajectories. (B) Normalized mean squared error on test data by time point after the 1st univariate PLS iteration. Normalized mean squared error versus time point on all genes, oocyte and sperm gene sets. Univariate PLS reaches lowest prediction error on oocyte gene set at late time points when oocyte gene expression peaks. Similarly, prediction error on sperm gene set is small at middle time points when sperm gene expression peaks. Each time-specific univariate PLS models the motif-expression correspondence for the gene set differentially expressed at the given time point. (0.40 MB TIF) [file pcbi.1000761.s003.tif]

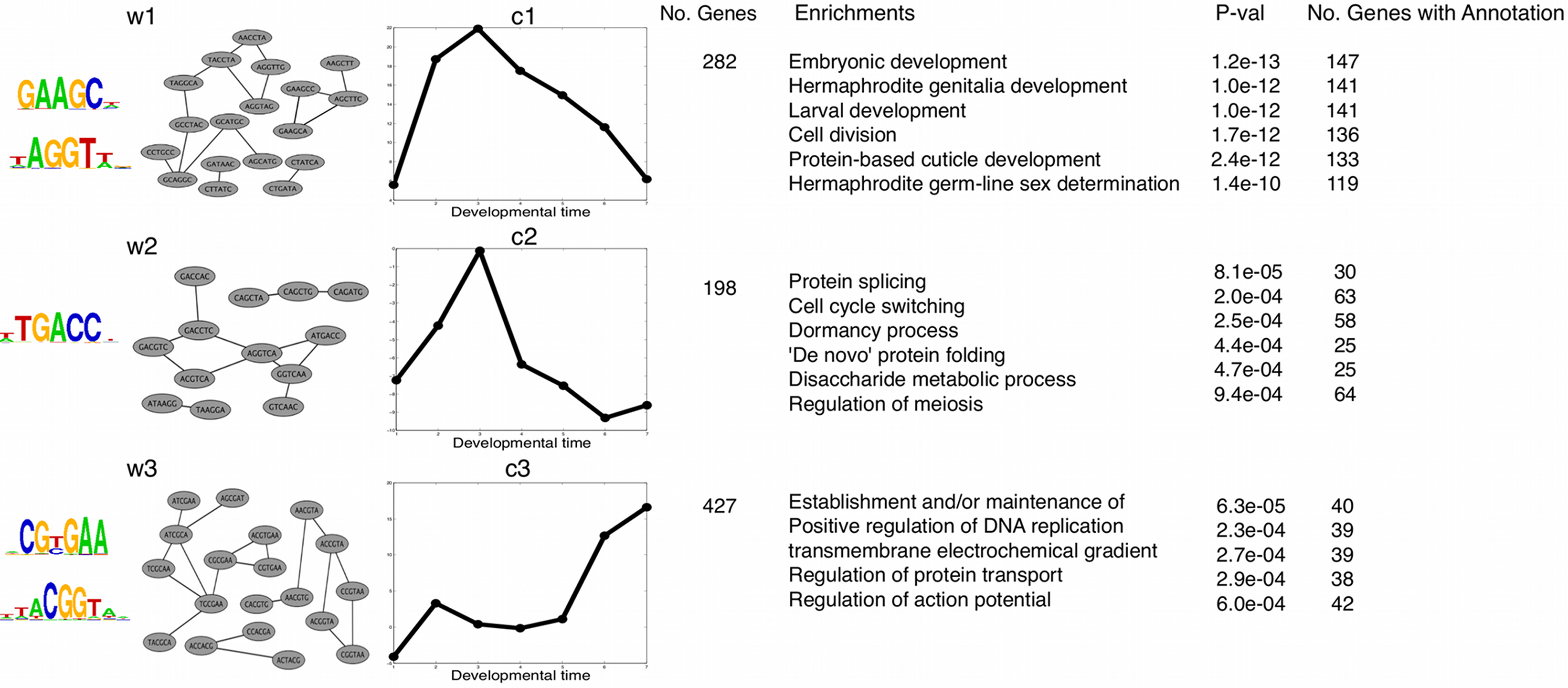

Supplement: Figure S4 — Latent factor analysis reveals graph-mers, expression patterns and significant associations of gene annotations. For each latent factor (i = 1…3), an associated mini graph-mer, extracted motif patterns and gene group are shown; annotations that are significantly enriched in each gene group are listed at the right (p<.001, uncorrected hypergeometric p-value), with p-values and number of genes associated with each annotation. (2.02 MB TIF) [file pcbi.1000761.s004.tif]

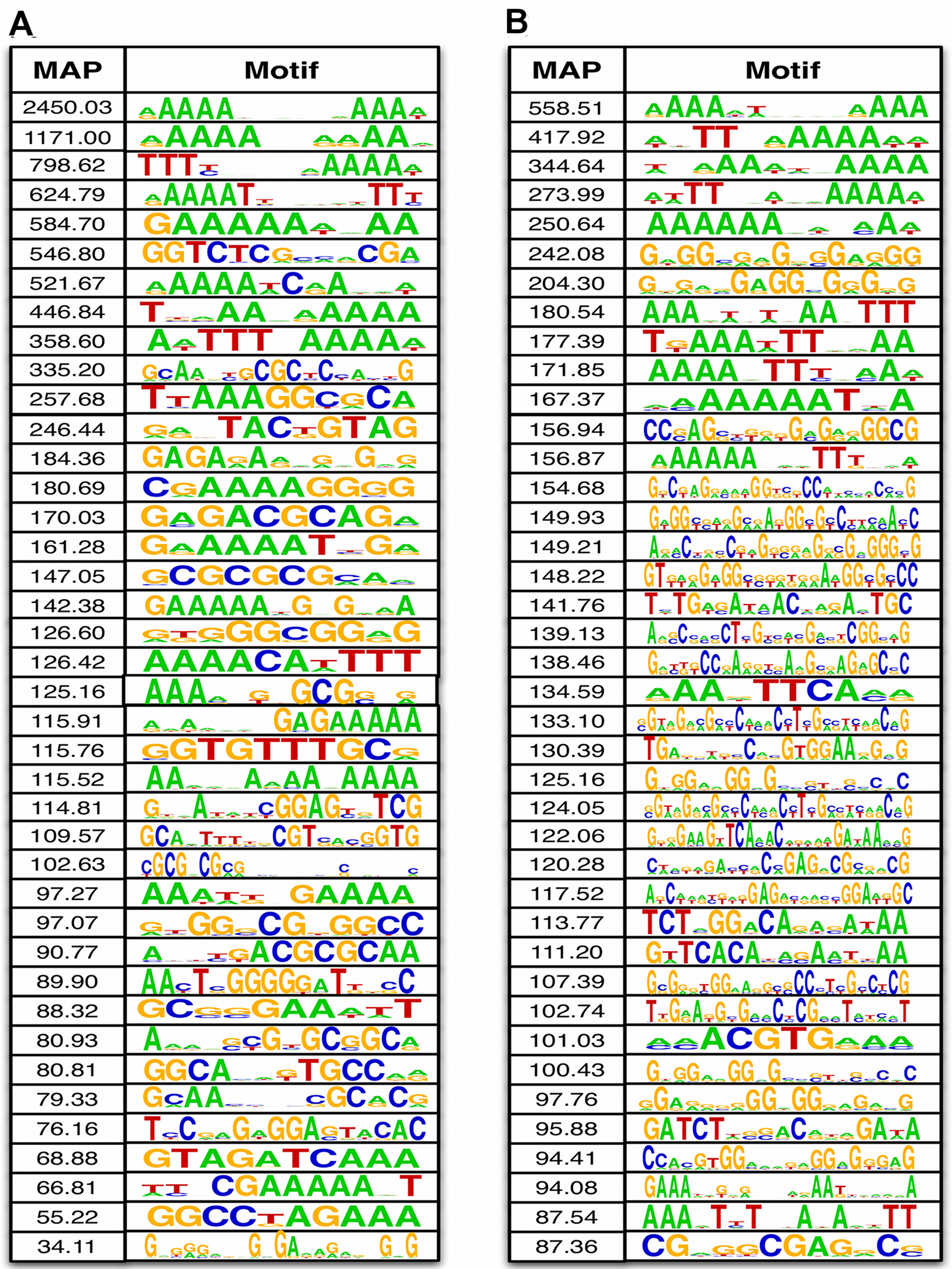

Supplement: Figure S5 — Motifs found by AlignACE in genes correlated with PC1 and PC2. (A) Top 40 AlignACE motifs in genes correlated with PC1 sorted by MAP score. Top ranked AA-rich and GG-rich motifs may result from low complexity regions, and several PCA motifs with relatively low MAP scores (e.g. MAP = 147.05, 90.77, 80.93) are similar to PLS 1st factor motifs. (B) Top 40 AlignACE motifs in genes correlated with PC2. Only one motif (MAP score = 101.03) is similar to our PLS sperm gene motif ACGTG from 2nd weight vector. None of the other PCA motifs matched any of the PLS 2nd factor motifs. (7.58 MB TIF) [file pcbi.1000761.s005.tif]

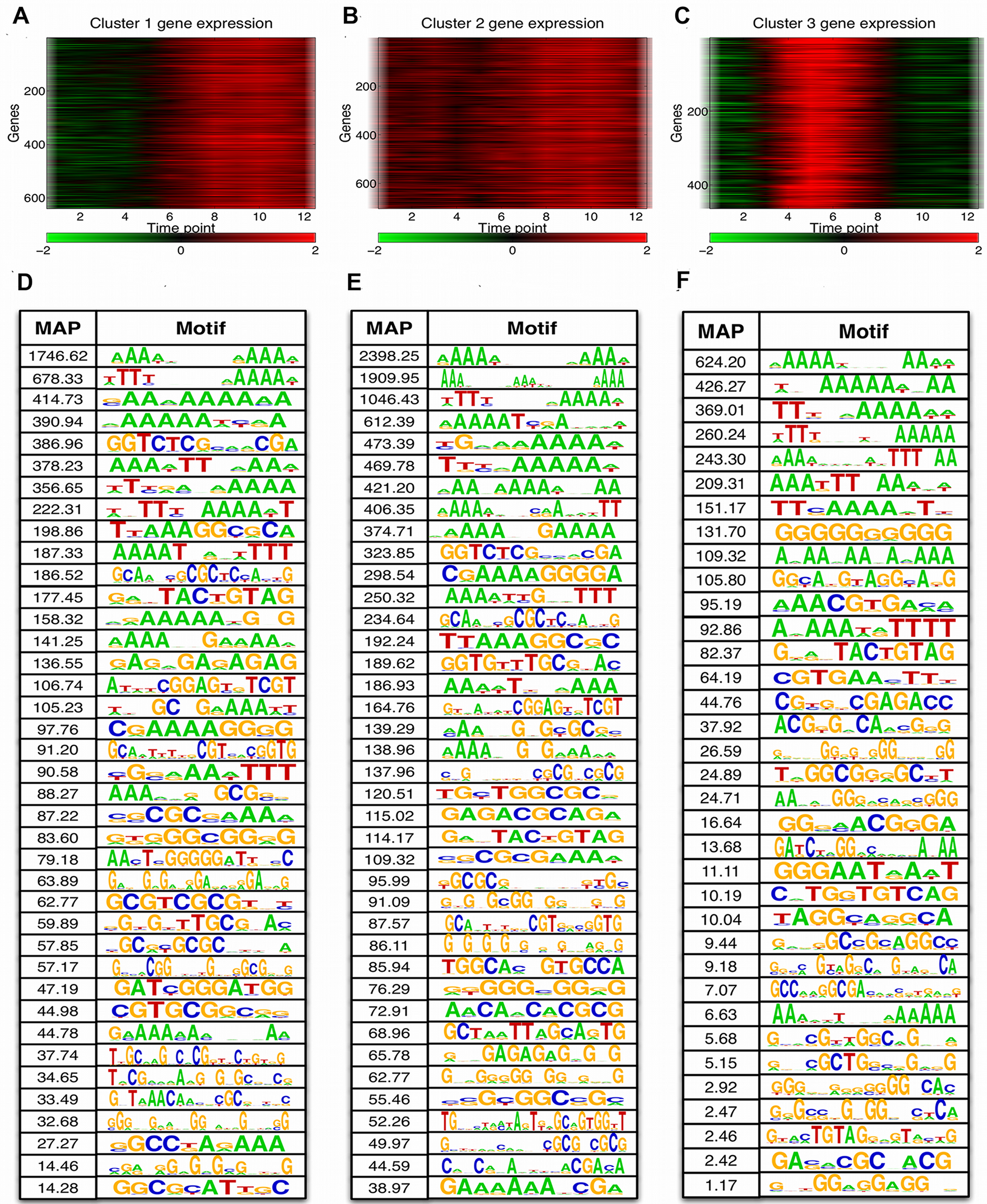

Supplement: Figure S6 — Motifs found by AlignACE in different gene clusters. (A) Expression patterns of genes in Cluster 1. (B) Expression patterns of genes in Cluster 2. (C) Expression patterns of genes in Cluster 3. (D) Top 40 AlignACE motifs found in Cluster 1 genes. (E) Top 40 AlignACE motifs found in Cluster 2 genes. (F) All 35 AlignACE motifs found in Cluster 3 genes. (10.87 MB TIF) [file pcbi.1000761.s006.tif]
