# Supplementary material for: Learning “graph-mer” Motifs that Predict Gene Expression Trajectories in Development
Source: PLoS Comput Biol. 2010 Apr 29;6(4):e1000761. doi: 10.1371/journal.pcbi.1000761 (PMC2861633; doi:10.1371/journal.pcbi.1000761)
Supplement: Text S1 — Supplementary results (0.08 MB PDF) [file pcbi.1000761.s007.pdf]

Learning "graph-mer" motifs that predict gene expression trajectories in development: supplementary results

Xuejing Li[1], Casandra Panea[2], Chris H. Wiggins[3] Valerie Reinke[2] Christina Leslie[4,*]
1 Department of Physics, Columbia University, New York, NY
2 Department of Genetics, Yale University, New Haven, CT
3 Department of Applied Physics and Applied Mathematics, Columbia University, New York, NY
4 Computational Biology Program, Sloan-Kettering Institute, New York, NY * E-mail: `cleslie@cbio.mskcc.org`

Gene set enrichment analysis for germline development

We have shown that the 1st and 2nd latent factors derived from graph-regularized PLS correspond to the expression patterns of the oocyte and sperm gene sets, respectively. We now use functional enrichment analysis to confirm that the genes identified statistically by these two factors are indeed enriched for oocyte or sperm genes. Given a gene set S and a real-valued ranking of all genes, we can use a procedure similar to gene set enrichment analysis (GSEA) [1] to establish whether the empirical cumulative distribution of genes in S is significantly shifted up or down compared to the set of genes not in S. Here, we use sperm and oocyte genes as gene sets and use either correlation with $\mathbf{c}_i$ or number of k-mer hits (for the top 50 graph-mer k-mers in $\mathbf{w}_i$) to produce the ranking. Figure S1A and S1B plot the empirical CDF of the correlation between gene expression and $\mathbf{c}_i$, $(i = 1, 2)$, showing that oocyte and sperm gene sets are enriched toward the top of the gene expression correlation. Similarly, Figure S1C and S1D plots the empirical CDF for k-mer hits, showing that oocyte and sperm gene sets are enriched in the corresponding k-mer hits. These results indicate that graph-regularized PLS can be used in conjunction with gene set analysis to identify functional categories that are supported both by shared motif information and expression trajectories.

k-mer enrichment in oocyte and sperm gene promoters

The top ranked k-mers we inferred from the first factor were characterized by rich CG content. To evaluate the statistical significance of those k-mers for oocyte genes, we studied the enrichment of all k-mers in oocyte genes. For each k-mer, a hypergeometric distribution p-value was estimated based on the counts of oocyte genes and all genes having the k-mer's presence. Figure S2A plots the hypergeometric distribution $-log_{10}(p\text{-value})$ representing k-mer enrichment in oocyte genes versus the k-mer's $\mathbf{w}_1$ value. We found a moderate correlation

(Pearson coefficient = 0.65) between the two variables, and in particular the k-mers highly ranked by $\mathbf{w}_1$ had p-values between 10^{-16} and 10^{-4}. This type of k-mer enrichment further validates the relevance of inferred k-mers from the first factor to oocyte genes. Similarly, we studied the enrichment of all k-mers in sperm genes and plotted the the hypergeometric distribution $-log_{10}(p\text{-value})$ representing k-mer enrichment in sperm genes versus the k-mer's $\mathbf{w}_2$ value (Figure S2B). There was some positive correlation between $-log_{10}(p\text{-value})$ and $\mathbf{w}_2$ (Pearson coefficient = 0.35), but it was weaker than that of oocyte genes.

Univariate PLS regression

In multivariate PLS, we found latent factors in motif space that explain gene expression trajectories over all time points simultaneously. For comparison, we also investigated learning motif information to predict time-specific gene expression by applying PLS to single experiment gene expression values, similar to existing regression-based algorithms like REDUCE [2]. We ran standard univariate PLS regression, where we trained and tested each time point separately. We learned up to five latent factors per time

point, giving a total of sixty factors. Figure S3 plots the normalized mean squared error on cross-validation test data versus number of PLS iterations for univariate and multivariate PLS . At each PLS iteration, univariate PLS learns twelve latent factors, corresponding to the twelve time points, while multivariate PLS learns one latent factor for all time points together. As shown in the Figure S3, univariate PLS achieves its lowest test error at the 1st iteration (12 latent factors), performing similarly though in fact marginally better than the best cross-validation error for standard multivariate PLS at the 4th iteration (4 latent factors). We conclude that learning motifs for each time point independently does as well as (indeed, slightly better than) the multivariate approach in terms of reducing squared error, but it does so at the cost of a more complex model.

We were also interested in evaluating univariate PLS on biological gene sets across time points, to see whether correlating motifs with certain time points can significantly explain the differential expression of the gene sets. We only examined the time-specific twelve factors corresponding to first latent factors for each time point, as univariate PLS starts overfitting after the 1st iteration. Figure S3 plots the time-specific normalized mean squared prediction error versus time point as the first twelve latent factors were applied to the twelve time points. We also estimated the prediction error on oocyte and sperm gene sets, and found that their prediction error profiles seemed to be anti-correlated with their expression profiles, respectively. Univariate PLS reaches lowest prediction error on oocyte gene set at late time points when oocyte gene expression peaks. Similarly, prediction error on sperm gene set is small at middle time points when sperm gene expression peaks. These results are expected, as each time-specific univariate PLS models the motif-expression correspondence for the gene set differentially expressed at the given time point.

Nonetheless, we found the k-mers ranked top by weight vectors at those middle or late time points to be fairly similar. This redundancy confirmed our earlier hypothesis that neighboring time points, either in the middle or late stages, are correlated and help us discern essentially the same motifs. Multivariate PLS reduces this type of redundancy in the model by learning fewer latent factors to map from motif to full expression patterns.

Latent factors for the worm life cycle

As a final test of our approach, we applied graph-regularized PLS to a full life cycle *C. elegans* developmental time course, consisting of whole-animal gene expression profiles from egg to adult [3]. The data set contains seven time points during the time course from oocyte to adulthood: one oocyte sample, one embryonic sample, four larval samples and one young adult sample. After removing genes exhibiting little variance in expression over time, we obtained the gene expression matrix for about 5500 genes. As before, we scanned the promoter sequences for candidate 6-mers and 7-mers and filtered k-mers based on expected counts in background sequences (see Methods).

Using 10-fold cross-validation on held-out genes for up to five latent factors, graph-regularized PLS achieved the lowest mean squared prediction error after the 3rd latent factor. To extract biological information from the algorithm output, we analyzed the first three factors to determine potentially coregulated gene groups and corresponding biological functions. We assigned each gene g to the gene group associated with a factor i based on $\mathbf{TU}$ values (see Methods), so that both the gene's promoter motif content and its expression pattern must be similar to the corresponding weight vectors for the latent factor. We then created three gene groups associated with the first three latent factors and studied significant enrichments of gene annotations ($p < 0.001$ by hypergeometric distribution). Figure S4 illustrates the mini graph-mers from $\mathbf{w}_i$, weight vectors $\mathbf{c}_i$, number of genes, top enriched gene annotations, p-values and number of genes associated with each annotation for the first three latent factors.

As shown in the figure, the 1st gene group is significantly annotated with the GO term germ-line sex differentiation, and in the corresponding mini graph-mer we again found the motif GATAA, the binding site recognized by the ELT-1 protein, which is highly expressed in the germ-line [4].

Motif discovery: comparison with principal component analysis

We performed principal component analysis (PCA) on the time series gene expression data and found that the principal components (PCs) are less smooth than their corresponding PLS weight vectors. To further compare PCA and PLS in terms of extracted motifs, we used AlignACE [5], a Gibbs sampling based motif finding algorithm, to discover motifs associated with the first two PCs, using the following procedure. First, we selected genes highly correlated with PC_1 and PC_2 (Pearson correlation coefficient ≥ 0.9) and obtained two gene sets consisting of 1248 and 415 genes for PC_1 and PC_2 respectively. Second, we ran AlignACE on the upstream regions of genes in each set, producing 58 motifs for PC_1 and 89 motifs for PC_2 in order of descending MAP scores, the metric for motif strength used by AlignACE. Figure S5A and S4B shows the two tables consisting of top 40 motifs for PC_1 and PC_2 respectively. In both tables, we see many AA-rich and GG-rich motifs that are highly ranked by MAP score, which likely come from low complexity sequence regions and probably do not represent biological binding sites. In Figure S5A for PC_1, AlignACE found several CG-rich motifs with relatively low MAP scores (e.g. MAP = 147.05, 90.77, 80.93). Among these motifs, most contain the core element CGCGC, matching the top ranked 50 k-mers of 1st PLS weight vector.

In Figure S5B for PC_2, only one motif (MAP score = 101.03) is similar to our PLS sperm gene motif ACGTG from the 2nd weight vector. However, it ranks low by MAP score and top ranked motifs seem to be background sequences with local AA or GG enrichment. These results suggest that we cannot fully retrieve the motifs learned by PLS simply by analyzing genes correlated with PC_1 and PC_2. Rather, PLS appears to recover more complete motif information by directly setting up a correspondence between promoter sequence and gene expression.

Motif discovery: comparison with clustering analysis

To show that PLS learns motifs that simple clustering based method does not find, we performed hierarchical clustering on genes using the Cluster 3.0 [6, 7] software package and ran the AlignACE program to discover overrepresented motifs in the gene clusters. In the hierarchical clustering step, we clustered genes by the similarity of their temporal expression profiles and determined gene clusters with distinct expression patterns. Using average linkage for calculation of cluster distance, we identified five large gene clusters within which the Pearson coefficient exceeds 0.80. In particular, we found three large gene clusters exhibiting expression patterns similar to oocyte or sperm gene expression, as illustrated in Figure S6. Genes in Cluster 1 display very low levels of expression early in the time course (time points 1 to 5) and then show an abrupt increase (time points 6 and 7). Meanwhile, genes in Cluster 2 have higher levels of expression at early time points and show a more gradual increase in expression over time. Genes in Cluster 3 are characterized by a sharp increase in expression at time points 3 and 4 and a sharp decline at time points 7 and 8, an expression pattern seen in many sperm genes. We applied AlignACE analysis on the three gene clusters and learned 47, 53 and 36 motifs for Clusters 1, 2 and 3 respectively. Figure S6 displays the three motif tables consisting of the top 40 motifs for Clusters 1 and 2 and all 35 motifs for Cluster 3, sorted in order of descending MAP scores. Similar to AlignACE on PCA gene sets, there are many AA-rich and GG-rich motifs that may come from low complexity sequence regions. For Cluster 1 and 2, which resemble the oocyte gene expression patterns, four motifs with relatively low MAP scores (MAP = 87.22, 57.85 with ranks 22, 28 among motifs in Cluster 1; and MAP = 109.32, 95.99 with ranks 24, 24 among motifs in Cluster 2) match PLS 1st weight vector motif CGCGC. Two motifs (MAP = 192.24, 120.51 at ranks 14, 21) in Cluster 2 contain the core element GGCGC found by PLS 1st weight vector. For Cluster 3, none of the AlignACE motifs match the top ones found by PLS 2nd weight vector.

References

1. Subramanian A, Tamayo P, Mootha V, Mukherjee S, Ebert B, et al. (2005) Gene set enrichment analysis: a knowledge-based approach for interpreting genome-wide expression profiles. Proceedings of the National Academy of Sciences 102: 15545–15550.

2. Bussemaker HJ, Li H, Siggia ED (2001) Regulatory element detection using correlation with expression. Nature Genetics 27: 167-171.

3. Hill A, Hunter C, Tsung B, Tucker-Kellogg G, Brown E (2000). Genomic analysis of gene expression in C. elegans.

4. Shim Y (1999) elt-1, a gene encoding a caenorhabditis elegans GATA transcription factor, is highly expressed in the germ lines with msp genes as the potential targets. MolCells 9: 535-541.

5. Hughes J, Estep P, Tavazoie S, Church G (2000) Computational identification of cis-regulatory elements associated with groups of functionally related genes in Saccharomyces cerevisiae. Journal of Molecular Biology 296: 1205–1214.

6. de Hoon M, Imoto S, Nolan J, Miyano S (2004) Open source clustering software. Bioinformatics 20: 1453.

7. Eisen M, Spellman P, Brown P, Botstein D (1998). Cluster analysis and display of genome-wide expression patterns.
